# TREX1 is required for microglial cholesterol homeostasis and oligodendrocyte terminal differentiation in human neural assembloids

**DOI:** 10.1038/s41380-023-02348-w

**Published:** 2023-12-21

**Authors:** Gabriela Goldberg, Luisa Coelho, Guoya Mo, Laura A. Adang, Meenakshi Patne, Zhoutao Chen, Ivan Garcia-Bassets, Pinar Mesci, Alysson R. Muotri

**Affiliations:** 1 Department of Pediatrics, School of Medicine, University of California San Diego, La Jolla, CA 92093 USA; 2 Department of Cellular & Molecular Medicine, School of Medicine, University of California San Diego, La Jolla, CA 92093 USA; 3 Biomedical Sciences Graduate Program, School of Medicine, University of California San Diego, La Jolla, CA 92093 USA; 4 Universal Sequencing Technology Corporation, Carlsbad, CA 92011 USA; 5 Division of Neurology, Children’s Hospital of Philadelphia, Philadelphia, PA USA; 6 Axiom Space, Houston, TX 77058 USA; 7 Kavli Institute for Brain and Mind, University of California San Diego, La Jolla, CA 92093 USA; 8 Center for Academic Research and Training in Anthropogeny (CARTA) and Archealization (ArchC), University of California San Diego, La Jolla, CA 92093 USA

**Keywords:** Cell biology, Neuroscience, Stem cells

## Abstract

Three Prime Repair Exonuclease 1 (*TREX1*) gene mutations have been associated with Aicardi-Goutières Syndrome (AGS) – a rare, severe pediatric autoimmune disorder that primarily affects the brain and has a poorly understood etiology. Microglia are brain-resident macrophages indispensable for brain development and implicated in multiple neuroinflammatory diseases. However, the role of TREX1 – a DNase that cleaves cytosolic nucleic acids, preventing viral- and autoimmune-related inflammatory responses – in microglia biology remains to be elucidated. Here, we leverage a model of human embryonic stem cell (hESC)-derived engineered microglia-like cells, bulk, and single-cell transcriptomics, optical and transmission electron microscopy, and three-month-old assembloids composed of microglia and oligodendrocyte-containing organoids to interrogate TREX1 functions in human microglia. Our analyses suggest that TREX1 influences cholesterol metabolism, leading to an active microglial morphology with increased phagocytosis in the absence of TREX1. Notably, regulating cholesterol metabolism with an HMG-CoA reductase inhibitor, FDA-approved atorvastatin, rescues these microglial phenotypes. Functionally, TREX1 in microglia is necessary for the transition from gliogenic intermediate progenitors known as pre-oligodendrocyte precursor cells (pre-OPCs) to precursors of the oligodendrocyte lineage known as OPCs, impairing oligodendrogenesis in favor of astrogliogenesis in human assembloids. Together, these results suggest routes for therapeutic intervention in pathologies such as AGS based on microglia-specific molecular and cellular mechanisms.

## Introduction

Three Prime Repair Exonuclease 1 (TREX1; OMIM 606609) is the most abundant mammalian exonuclease that degrades cytoplasmic DNA in a 3′–5′ manner [1, 2], aiding in antiviral defense and preventing autoimmunity [3–5]. Mutations in *TREX1* have been associated with severe cases of Aicardi-Goutières Syndrome (AGS; OMIM 225750) [6] – a rare autosomal recessive, multi-system autoinflammatory disorder that affects development [7, 8]. Early-onset brain degeneration related to TREX1 results in severe intellectual and physical disability [6, 9–12]. Radiographic features of AGS patients have uncovered leukodystrophy symptoms such as white matter rarefaction, deep white matter cysts, and inadequate myelination in patients with *TREX1* mutations [13]. *Trex1*-knockout (KO) mouse models have helped decipher mechanisms for systemic autoimmune pathology in AGS, but do not recapitulate the neurological aspects [14], leaving open the possibility that mice do not represent a good model for this condition. Together with the difficulties associated with studying human brain disorders, new human models are necessary to understand the neuropathology of AGS.

Using a pluripotent stem cell (PSC)-derived in vitro human brain development model, we previously showed that *TREX1*-KO astrocytes upregulate interferon-stimulated genes (ISGs) and secrete type I interferons (IFNs) and cytokines that drive neurotoxicity due to the cytosolic accumulation of Long Interspersed Element-1 (L1) retrotransposons [15]. However, it remains unknown the role of other brain cells necessary for orchestrating brain development such as microglia [16]. Microglia – the brain-resident immune glia – play a pivotal role in brain development by engulfing dead cells, dysfunctional synapses, and myelin debris [17–19]. During aging and disease, microglia become less homeostatic and show profound functional changes such as increased secretion of inflammatory cytokines, altered morphology, and accumulation of lysosomal deposits indicative of impaired phagocytosis [20, 21]. Recent single-cell RNA sequencing (scRNA-seq) studies have revealed disease-specific states of microglia, including disease-associated microglia (DAM), neurodegenerative microglia (MGnD), and lipid-droplet accumulating microglia (LDAM) [22–24]. Lipid-laden or foamy macrophages/microglia have also been observed in postmortem brain tissues of patients with multiple sclerosis (MS) and mouse models of MS, leading to impaired remyelination and intermediate inflammatory states, suggesting that microglial lipid metabolism is essential for mitigating inflammatory myelinating diseases [25–27]. Microglia, the brain-resident immune cells, colonize the developing brain prior to neurogenesis and are involved in several important brain processes including myelinogenesis, synaptic plasticity, and the pruning of synapses [19, 28, 29].

Extensive research has been conducted linking innate inflammatory pathways to lipid metabolism in macrophages during viral infection [30–33]. The role of microglial lipid metabolism in neurodegenerative diseases such as MS, Alzheimer’s Disease (AD), and neuropathic pain have recently come to the forefront as potential therapeutic targets and research areas of interest [25, 34–36]. However, the relationship between microglial homeostasis and developmental myelination has not been studied in the context of neurodevelopmental inflammatory disorders such as AGS. Recent advances in hPSC-derived three-dimensional (3D) regionalized neural organoids allow researchers to model genetic hypomyelinating neurodevelopmental disorders [37, 38]. Microglia, however, derive from the yolk sac and do not appear within regionalized neural organoids, which are derived from neuroectoderm signaling, meaning the study of neuro-immune interactions in a human developmental context requires the addition of microglia from an outside source [38].

Here, we identify a state of developmental microglia with a unique transcriptional signature, impaired cholesterol metabolism, an active amoeboid morphology, and increased phagocytosis rates – all of which are L1 independent, an astrocytic *TREX1*-related feature. We also develop a 3D assembloid model to study glial interactions in a cellular model of embryonic brain development revealing that *TREX1*-KO microglia disrupt an intermediate step in the oligodendrocyte lineage—i.e., the pre-oligodendrocyte progenitor cell (pre-OPC) to OPC progression—indicating the possibility of microglia-driven pathologies in AGS.

## Results

### *TREX1*-KO microglia demonstrate an active phenotype

**Fig. 1:**
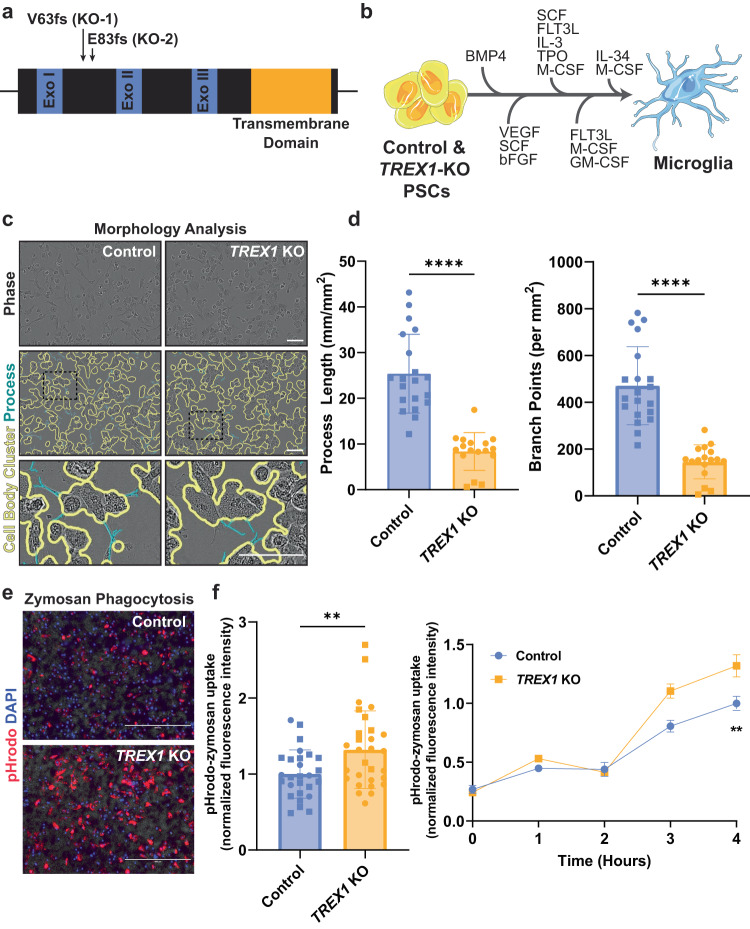
*TREX1*-KO microglia demonstrate an active phenotype. **a** Schematic representation of the *TREX1* gene showing the mutations in the pluripotent lines. “Exo” is short for exonuclease domain. **b** Schematic representation of the protocol used to differentiate control and *TREX1*-KO PSCs to microglia-like cells. Full description of the differentiation protocol is in the methods. **c** Representative brightfield images of microglia-like cells showing an overlay of outlines depicting cell-body clusters and cell processes determined using the Incucyte S3 live-cell analysis system. Scale bar is 100 µm. **d** Left: Quantification of process length to analyze microglial morphology. Right: Quantification of number of branch points. The presented values are means ± SEM (Control: *n* = 20 wells; *TREX1*-KO: *n* = 17 wells). **e** Representative images of microglia 4 h post pHrodo-zymosan phagocytosis assay. Scale bars are 400 µm. **f** Left: Four hour pHrodo-zymosan uptake normalized fluorescence intensity. Right: Line graph of pHrodo-zymosan uptake over 4 h. The presented values are means ± SEM (Control: *n* = 27 wells; *TREX1*-KO: *n* = 29 wells). Symbols in bar graphs indicate cell lines used: circles, Control-1 and *TREX1*-KO-1 pair; squares, Control-2 and *TREX1*-KO-2 pair. Colors in graphs represent either the control lines (blue) or the *TREX1*-KO lines (yellow). *p < 0.05, **p < 0.01, ***p < 0.001, ****p < 0.0001.

To understand TREX1 functions in microglial biology, we leveraged CRISPR/Cas9-engineered H9 hESC lines that we previously developed carrying single nucleotide insertions in homozygosis that cause an early stop codon at amino acid 100 owing to frameshift mutations starting at the amino acids valine 63 (V63) and glutamate 83 (E83): TREX1-KO-1 (V63fs) and TREX1-KO-2 (E83fs), respectively (Fig. 1a) [15]. To serve as isogenic controls to these lines, two other H9 hESC-derived and clonally expanded lines that underwent CRISPR/Cas9 endonuclease cleavage but do not carry *TREX1* mutations were chosen. These control lines were named Control-1 (WT63) and Control-2 (WT83).

To generate microglia-like cells, we supplemented PSCs with a cocktail of differentiation factors in four sequential steps [39] (Fig. 1b). In the last step, microglial precursors are harvested from the media, allowed to adhere to uncoated tissue culture dishes, and supplemented with a final cocktail of factors for one additional week to allow the cells to reach a microglia-like state. The resultant microglia-like cells express typical microglial markers such as IBA1, TREM2, CX3CR1, and P2Y12R proteins (Fig. S1A). In addition, more than 90% of microglia-like cells were double positive for additional typical microglial surface markers CD45 and CD11b (Fig. S1B). Thus, we concluded that the engineered and control microglia cultures were suitable for assessing neuroinflammation in the context of *TREX1*-KO development.

To determine if *TREX1*-KO microglia show an increased expression of L1 retrotransposons, similar to what we previously observed in *TREX1*-KO astrocytes [15], we performed real-time (RT) quantitative (q)PCR using three different primers corresponding to different regions of L1 and found no statistically significant difference between control and *TREX1*-KO microglia (Fig. S1C). We then performed an ELISA on microglia conditioned media (MCM) to determine if *TREX1*-KO microglia are secreting an excess of type I IFNs and found no difference between *TREX1*-KO MCM and control MCM for IFNα-2a and IFN*β* (Fig. S1D), suggesting that *TREX1*-KO microglia may not share similar inflammatory phenotypes to *TREX1*-KO astrocytes previously observed [15].

Microglia uniquely adapt to the surrounding environment and show dynamic morphologies ranging from ramified processes with small cell bodies during surveillance to large amoeboid cells when actively phagocytosing or during inflammation [40, 41]. To determine potential differences in microglial morphology, we measured process lengths and branch points from brightfield images of microglia. These measurements revealed that *TREX1*-KO microglia have shorter process lengths and fewer branch points than control cells (Fig. 1c, d), suggesting that *TREX1*-KO microglia have a less ramified morphology. Since microglia are derived from yolk sac macrophage precursor cells, their main function, like other tissue macrophages, is to perform phagocytosis in the brain [18, 35, 42]. Using pHrodo-conjugated zymosan particles, we determined that *TREX1*-KO microglia have more fluorescent particles after zymosan supplementation compared to control (Fig. 1e, f), suggesting that *TREX1*-KO microglia have an increased rate of phagocytosis. Taken together, these data indicate that *TREX1*-KO microglia show morphological differences and increased phagocytosis compared to controls, but may not have a *TREX1*-KO astrocyte-like inflammatory phenotype.

### Cholesterol synthesis is dysregulated in *TREX1*-KO microglia

**Fig. 2:**
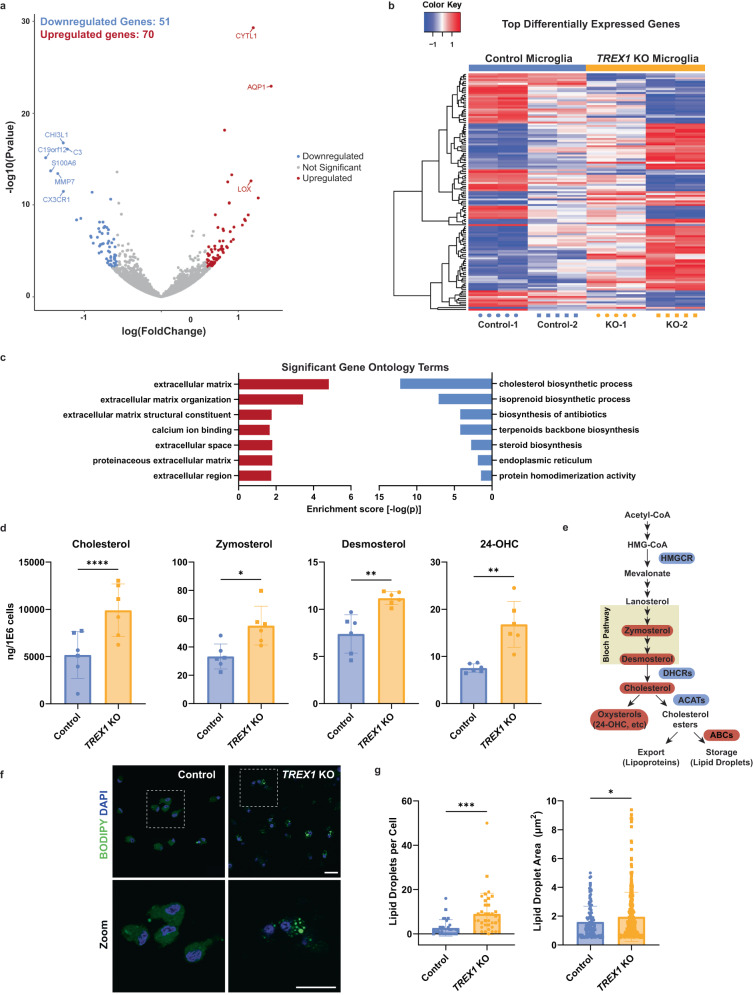
Cholesterol synthesis is dysregulated in *TREX1*-KO microglia. **a** Volcano plot showing differentially expressed genes between control and *TREX1*-KO microglia obtained through RNA sequencing. Red dots are genes significantly upregulated in *TREX1*-KO microglia, blue dots are significantly downregulated genes, and gray dots are not significant. **b** Heatmap of the top differentially expressed genes between control and *TREX1*-KO microglia. Red are upregulated genes and blue are downregulated genes. Rows are clustered using the Euclidean distance method. **c** Bar graph showing the top significant gene ontology terms. Red bars are upregulated in *TREX1*-KO microglia and blue bars are downregulated. **d** Total sterol/oxysterol analysis of microglia showing increased zymosterol, desmosterol, and 24-OHC in *TREX1*-KO microglia. The presented values are means ± SEM (*n* = 12; 3 independent microglia differentiation experiments and 4 lines). **e** Simplified schematic of the cholesterol biosynthesis process showing increases (red) in immediate precursors and oxysterol, and changes (blue are downregulated and red are upregulated) in the expression of genes associated with cholesterol enzymes in *TREX1*-KO microglia. **f** Representative images of BODIPY-stained microglia. Scale bars are 20 µm. **g** Left: Quantification of number of lipid droplets per cell. Right: Quantification of lipid-droplet area. The presented values are means ± SEM (*n* = 60 cells). Symbols in bar graphs indicate cell lines used: circles, Control-1 and *TREX1*-KO-1 pair; squares, Control-2 and *TREX1*-KO-2 pair. Colors in graphs represent either the control lines (blue) or the *TREX1*-KO lines (yellow). *p < 0.05, **p < 0.01, ***p < 0.001, ****p < 0.0001.

To further characterize *TREX1*-KO microglia and investigate related mechanisms for our observations, we performed bulk RNA sequencing (Supplementary Table S1). We hypothesized that *TREX1*-KO microglia would have upregulated ISGs and other inflammatory genes, similar to what are normally observed in peripheral blood mononuclear cells (PBMCs) of AGS patients [43, 44]. Our experiment yielded 121 differentially expressed genes between control and *TREX1*-KO microglia with a false discovery rate (FDR) < 0.05 and a fold change (FC) > 1.5 (Fig. 2a, b). Surprisingly, we observed a downregulation in microglial homeostasis genes such as *CX3CR1* and *C3* (Fig. 2a), a similar phenotype found in disease-associated microglia (DAMs) which are characterized by increased neuroinflammation and phagocytosis [22, 35]. We then performed gene ontology analysis and discovered a downregulation in genes associated with the cholesterol biosynthetic process (Figs. 2c, S2A, and S2B). We confirmed these findings through RT-qPCR of several cholesterol biosynthesis genes such as *HMGCR*, *DHCR7*, *DHCR24*, *ACAT1*, *ACAT2*, and *ABCA1* (Fig. S2C). Therefore, *TREX1*-KO microglia are transcriptionally different from control microglia with downregulated microglial homeostasis and cholesterol biogenesis genes, suggestive of a proinflammatory disease phenotype.

Microglial cholesterol metabolism has been implicated in AD, demyelinating diseases, neuropathic pain, and aging [24, 25, 34, 35, 45]. In this context, we collected blood lipid profiles of patients with molecularly confirmed AGS and found abnormal lipid profiles in a subset of patients (Fig. S2E). Specifically, triglycerides were elevated (*n* = 21/44, including *n* = 2/7 within the TREX1 subcohort) and high-density lipoprotein (HDL) cholesterol levels were low (*n* = 17/42, including *n* = 4/6 of the TREX1 subcohort). Cholesterol metabolism is a tightly regulated process that, when perturbed, initiates an innate immune feedback loop producing inflammation [32, 33]. We hypothesized that the downregulation in cholesterol biosynthesis genes observed in *TREX1*-KO microglia was due to an accumulation of intracellular lipids, similar to what has been observed in proinflammatory microglia of the aging brain [24]. We performed a total sterols lipidomic panel and found increased concentrations of cholesterol between control and *TREX1*-KO microglia (Figs. 2d, e and S2D). Immediate cholesterol precursors, zymosterol and desmosterol, as well as the brain-specific oxysterol, 24-OHC, were also increased in *TREX1*-KO microglia (Fig. 2d, e).

Accumulation of desmosterol has been associated with inflammation in foamy macrophages of atherosclerotic lesions and in microglia associated with demyelinated lesions and in the aging brain [24, 25, 32]. Interestingly, foamy macrophages have been described in a case of AGS [46]. To confirm the presence of foamy-like microglia, we performed a BODIPY™ stain – a fluorescent dye that stains for neutral lipids and is commonly used to label intracellular lipid droplets [24] – and discovered that *TREX1*-KO microglia have more lipid droplets per cell and those droplets are bigger than control microglia (Fig. 2f, g). These data suggest that TREX1 may be involved in regulating cholesterol metabolism and intracellular lipid accumulation in microglia.

### Atorvastatin rescues cholesterol-associated and active *TREX1*-KO microglia phenotype

**Fig. 3:**
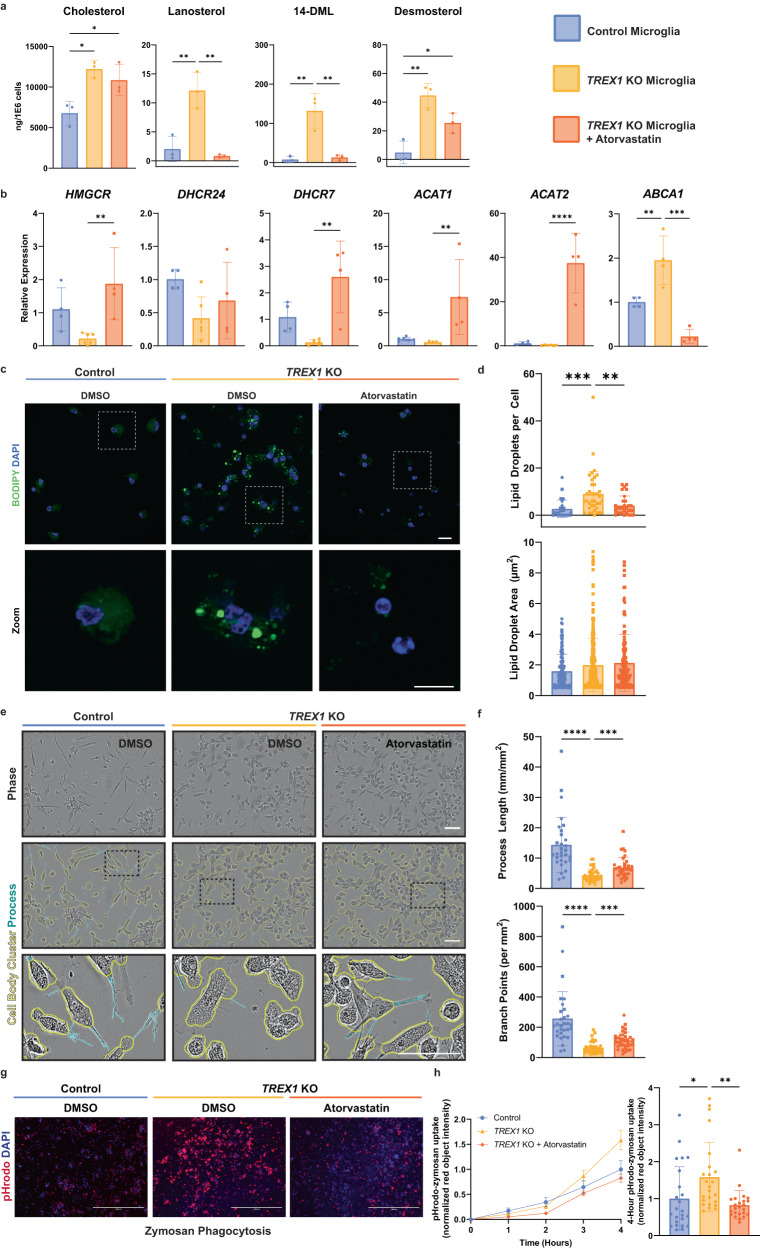
Atorvastatin rescues cholesterol-associated and active *TREX1*-KO microglia phenotype. **a** Total sterol/oxysterol analysis of atorvastatin-treated microglia showing rescue of lanosterol, 14-DML, and partial rescue of desmosterol. The presented values are means ± SEM (*n* = 12; 3 independent microglia differentiation experiments and 4 lines). **b** Relative expression (RT-qPCR) of cholesterol synthesis genes (*HMGCR*, *DHCR7*, *DHCR24*, *ACAT1*, *ACAT2*, and *ABCA1*) in atorvastatin-treated microglia. The presented values are means ± SEM (*n* = 16; 2 independent experiments and 4 cell lines). **c** Representative images of BODIPY-stained atorvastatin-treated microglia. Scale bars are 20 µm. **d** Left: Quantification of number of lipid droplets per cell. Right: Quantification of lipid-droplet area. The presented values are means ± SEM (*n* = 30 cells per condition). **e** Representative brightfield images of atorvastatin-treated microglia showing an overlay of outlines depicting cell-body clusters and cell processes determined using the Incucyte S3 live-cell analysis system. Scale bar is 100 µm. **f** Left: Quantification of process length to analyze microglial morphology. Right: Quantification of number of branch points. The presented values are means ± SEM (*n* = 30 wells per condition). **g** Representative images of atorvastatin-treated microglia 4 h post pHrodo-zymosan phagocytosis assay. Scale bars are 1000 µm. **h** Left: Line graph of pHrodo-zymosan uptake over 4 h. Right: Four hour pHrodo-zymosan uptake normalized fluorescence intensity. The presented values are means ± SEM (*n* = 24 wells per condition). Symbols in bar graphs indicate cell lines used: circles, Control-1 and *TREX1*-KO-1 pair; squares, Control-2 and *TREX1*-KO-2 pair. Colors in graphs represent the control lines (blue), *TREX1*-KO lines (yellow), or *TREX1*-KO lines treated with atorvastatin (orange). *p < 0.05, **p < 0.01, ***p < 0.001, ****p < 0.0001.

Normalizing cholesterol metabolism in microglia has been proposed as a method for treating neurodegenerative diseases such as AD and inflammatory diseases such as MS and neuropathic pain [34, 47–51]. To determine if PSC-derived microglia are receptive to cholesterol-normalizing drugs, we matured *TREX1*-KO microglia in the presence of atorvastatin – a commonly used lipid-lowering statin medication that inhibits HMG-CoA reductase and prevents cholesterol synthesis. We performed a total sterols lipidomic panel and observed a rescue in cholesterol intermediates lanosterol, 14-demethyl-lanosterol (14-DML), and desmosterol upon treatment with atorvastatin (Fig. 3a). We also observed a rescue in the expression of cholesterol metabolism genes such as *HMGCR*, *DHCR7*, *ACAT1*, *ACAT2*, and *ABCA1* with atorvastatin treatment (Fig. 3b). Atorvastatin treatment also rescued the accumulation of intracellular lipid droplets in *TREX1*-KO microglia (Fig. 3c, d). These results suggest that atorvastatin is an effective method for treating the dysregulated cholesterol synthesis observed in *TREX1*-KO microglia.

Next, we investigated whether rescuing cholesterol synthesis also rescues the morphology and phagocytosis of *TREX1*-KO microglia. Treatment with atorvastatin increased process length and branch points in *TREX1*-KO microglia (Fig. 3e, f), rescuing morphology. Atorvastatin treatment also decreased phagocytosis of zymosan bioparticles (Fig. 3g, h), restoring phagocytosis rates to control levels. These data suggest that microglial activation status depends on cholesterol synthesis, and normalizing cholesterol regulation with atorvastatin can restore microglia from an activated state to a more resting- and surveillance-like state.

Type I IFN signaling and cholesterol metabolism form an immuno-metabolic circuit in macrophages [33]. Given our previous findings in *TREX1*-KO astrocytes, we next investigated whether type I IFN signaling triggered cholesterol dysregulation in microglia. We treated control microglia with polyinosine-polycytidylic acid [poly (I:C)] – a synthetic analog of double-stranded RNA that acts as a TLR3 agonist and triggers a type I IFN response [52]. Control microglia treated with poly (I:C) showed no changes in lipid profile or lipid-droplet accumulation (Fig. S3A, S3C, and S3D), suggesting that type I IFN signaling does not affect cholesterol metabolism in PSC-derived microglia. However, control microglia treated with poly (I:C) had a more activated morphology with shorter process lengths and fewer branch points (Fig. S3E and S3F), as well as severely decreased phagocytic activity of zymosan bioparticles (Fig. S3G and S3H). This suggests that *TREX1*-KO-associated microglial phenotypes may be independent of type I IFN signaling in PSC-derived microglia.

Because of the role of L1 in AGS pathology, reverse transcriptase inhibitors (RTis) have been trialed in AGS patients and in vitro studies where they rescue neurotoxic phenotypes in *TREX1*-KO astrocytes [15, 53]. Here, we tested if RTis would improve phenotypes in microglia. We matured *TREX1*-KO microglia in the presence of two RTis, 3TC and D4T, and observed no differences in lipid profile or lipid-droplet accumulation (Fig. S3A, S3C, and S3D). Microglia treated with RTis showed increased expression of *ACAT1*, *ACAT2*, and decreased expression of *ABCA1* (Fig. S3B), indicating that cholesterol transport and cholesterol ester conversion are restored to control levels with RTi treatment but lipid profiles or lipid-droplet accumulation are not affected. Further, there was no difference between DMSO- and RTi-treated *TREX1*-KO microglial morphology (Fig. S3E and S3F). Zymosan phagocytosis decreased in RTi-treated compared to DMSO-treated *TREX1*-KO microglia (Fig. S3G and S3H), however, suggesting that RTis partially rescue disease phenotypes of *TREX1*-KO microglia through a pathway that does not depend on type I IFN signaling or cholesterol metabolism.

### TREX1 in microglia is necessary for oligodendrogenesis

**Fig. 4:**
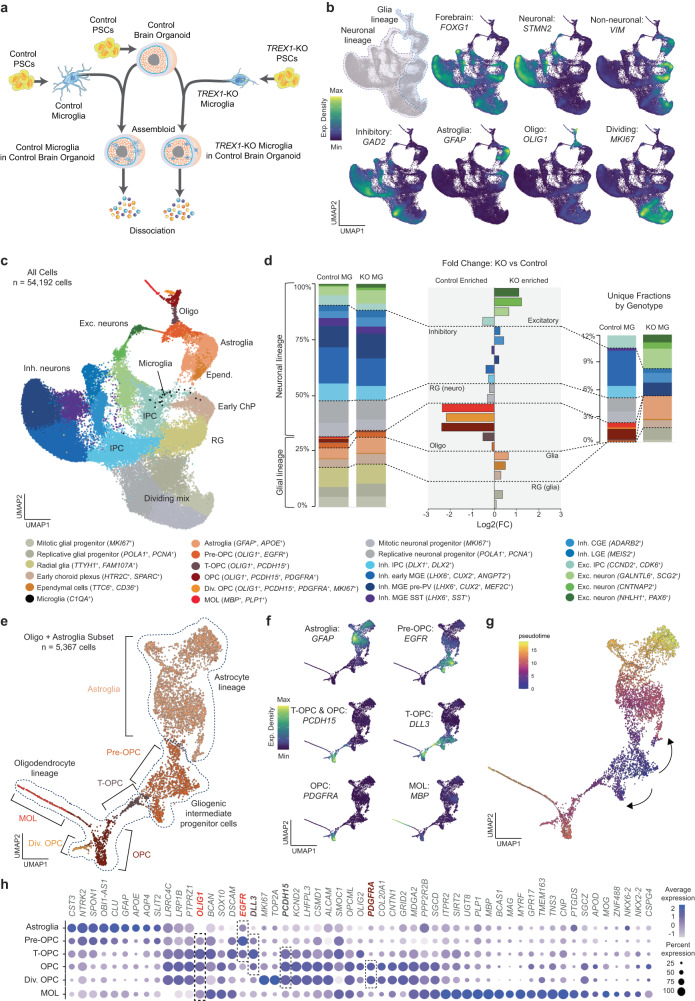
TREX1 in microglia is necessary for the development of myelinating oligodendrocytes. **a** Schematic representation of the assembloid experimental design. Differentiation protocols were performed in parallel prior to combining cells for assembloid generation. **b** Expression density of selected markers on Uniform Manifold Approximation and Projection (UMAP) plots showing integrated scRNA-Seq from 3 libraries of assembloids composed of control organoids and control microglia and 2 libraries of assembloids composed of control organoids and *TREX1*-KO microglia (in total, 54,192 cells). **c** Cell annotations on UMAP plot based on scRNA-seq data. **d** Left: Percentage distribution of cell types by conditions. Center: Fold change (log2) of percentage distribution between microglia genotype. Right: As in left but showing only unique fractions by microglia genotype. Cells color-coded in all graphs as in (left). The largest difference (by fold change) between microglia genotypes corresponds to oligodendrocyte cell types (MOLs, dividing OPCs, OPCs > t-OPCs > pre-OPCs). The largest differences (by quantity) correspond to a gain of excitatory and astroglia populations and a loss of inhibitory subtypes with mutant microglia. Radial glia with neuronal fate shifted to radial glia with glial fate. **e** Re-analysis of oligodendrocyte lineage and astroglia on UMAP plot with annotated cell types as indicated. **f** Expression density of selected markers. **g** Pseudotime analysis of oligodendrocyte lineage and astroglia starting at pre-OPCs. **h** Dot plot showing expression of selected marker genes in the five subpopulations of the oligodendrocyte lineage and astroglia. Dot sizes are the percentages of cells in each subpopulation that have detectable expression for the corresponding gene. Most relevant markers highlighted: *EGFR* primarily for pre-OPC and t-OPC; *DLL3* primarily for t-OPC; *PDCH15* for OPCs; *PDGFRA* for OPCs except t-OPCs. All cells express *OLIG1*. Dot color intensity represents average gene expression. MG: microglia; Oligo: oligodendrocyte; Astro: astrocyte; Micro: microglia; Exc.: excitatory; Inh.: inhibitory; Div.: dividing; RG: radial glia; IPC: intermediate progenitor cells; ChP: choroid plexus; OPC: oligodendrocyte progenitor cells; MGE: medial ganglionic eminence; CGE: caudal ganglionic eminence; LGE: lateral ganglionic eminence; PSCs: pluripotent stem cells.

Amoeboid microglia have been shown to engulf OPCs during brain development and regulate myelination [54]. Given clinical findings of white matter calcifications and abnormalities in AGS patients [6, 13], we hypothesized that *TREX1*-KO microglia impair myelination. To study this question, we developed an assembloid system using PSC-derived microglia and regionalized neural organoids. Following a protocol that generates organoids for the study of oligodendrocyte development, myelination, and interactions with other major brain cell types, known as human oligodendrocyte spheroids (hOLS) [37], we derived control hOLS and both control and *TREX1*-KO microglia in parallel (Fig. 4a). After one month of organoid differentiation, we combined microglial precursors with hOLS and continued culturing for a total of three months prior to profiling assembloids using scRNA-seq. These assembloids express the forebrain marker *FOXG1*, a large number of inhibitory neurons (*GAD2*+), astroglia (*GFAP*+), and oligodendrocyte lineage cells (*OLIG1*+) (Fig. 4b), as expected [37].

Using molecular markers for cell identity, we annotated the cells on a UMAP plot (Figs. 4c and S4A). When comparing the abundance of each cell type by microglia genotype, we observed the largest differences among the cells in the oligodendrocyte lineage, specifically OPCs, dividing OPCs, and myelinating oligodendrocytes (MOLs) (Fig. 4d). By number, inhibitory neurons also decreased whereas astroglia and excitatory neurons increased (Fig. 4d). The decrease in MOLs was confirmed by fluorescent staining of MBP^+^ cells and transmission electron microscopy showing early myelination ultrastructures of assembloids containing control microglia, but not in assembloids containing *TREX1*-KO microglia (Fig. S4B and S4C, respectively).

Within the oligodendrocyte lineage, we identified at least five populations which were re-clustered in combination with astroglia suggesting two differentiation trajectories starting from pre-OPCs as inferred by pseudotime analysis (Fig. 4e–g): pre-OPCs (*OLIG1*^*+*^*EGFR*^*+*^*PCDH15*^*-*^*PDGFRA*^*-*^), transitioning OPCs or t-OPCs (*OLIG1*^*+*^*EGFR*^*+*^*PCDH15*^*+*^*PDGFRA*^*-*^), OPCs (*OLIG1*^*+*^*EGFR*^*+*^*PCDH15*^*+*^*PDGFRA*^*+*^), dividing OPCs (*OLIG1*^*+*^*EGFR*^*+*^*PCDH15*^*+*^*PDGFRA*^*+*^*MKI67*^*+*^), and MOLs (*OLIG1*^*+*^*EGFR*^*+*^*PCDH15*^*+*^*PDGFRA*^*+*^*MBP*^*+*^).

These results suggest that microglial expression of TREX1 is necessary for normal progression of the oligodendrocyte lineage, and that the absence of TREX1 in microglia alone can disrupt the pre-OPC to t-OPC transition, depleting the assembloids of a fraction of t-OPCs, and most OPCs, dividing OPCs, and MOLs.

## Discussion

In this study, we identified a novel state of developmental microglia in AGS, which are L1 independent, and are defined by impaired cholesterol metabolism, lipid-droplet accumulation, an active amoeboid morphology, and increased rates of phagocytosis. The *TREX1*-associated microglial phenotype was rescued to control conditions upon treatment with atorvastatin – an FDA-approved HMG-CoA reductase inhibitor commonly used to treat hypercholesterolemia. Rescue of this phenotype was not achieved with RTis, and control microglia treated with poly(I:C) did not recapitulate the phenotype, suggesting that this microglial state is L1- and type I IFN-independent. We also characterized the role of microglial TREX1 in a cellular model of human brain development. Our development of a novel assembloid model where neurons, astrocytes, oligodendrocytes, and microglia are combined in a single PSC-derived assembloid made this discovery possible.

The advent of single-cell technologies, such as scRNA-seq and single-cell mass spectrometry, have shed light on the existence of heterogeneous microglial states in the mouse and human brain. Brain pathologies, such as neurodegeneration, transform relatively homogeneous homeostatic microglia into disease-related microglial states such as DAMs, MGnDs, and LDAMs in the adult mouse brain [22–24]. More recently, a subset of microglia that form nodules in aging white matter were identified and termed white matter-associated microglia (WAMs) [55]. Characterized by the activation of genes implicated in phagocytic activity, lipid metabolism, and partial activation of the DAM program, WAMs closely resemble the AGS microglial state we identified. Although WAMs were discovered in the aging brain, it is speculated that there exists an activation state continuum between WAMs and DAMs where both signatures can coexist in diseases such as AD [55, 56]. We hypothesize this same continuum may be associated with pathology in AGS. Another microglial subset discovered in postnatal white matter, which shares characteristics with our model, are proliferative-region associated microglia (PAMs) [57]. PAMs are characterized by an amoeboid morphology, a DAM signature, and the phagocytosis of newly formed oligodendrocytes. PAMs transiently appear in developing white matter and are thought to maintain tissue homeostasis for the development of evenly spaced out oligodendrocytes [57, 58]. Rather than appearing transiently during development, we speculate that AGS-associated microglia are constitutively present and inhibit the development of healthy oligodendrocytes. This could result in the extensive white matter disease that defines AGS. Further studies are needed to support this hypothesis.

Cholesterol metabolism plays a critical role in the pathogenesis of disease, with atherosclerosis being the most well-studied example [59, 60]. Dysfunctional cholesterol metabolism has also been linked to inherited autoimmune diseases such as mevalonate kinase (MVK) deficiency [61]. In MVK deficiency – a rare autoimmune disorder caused by mutations in the *MVK* gene – mevalonate, a cholesterol precursor, accumulates in patient macrophages and induces heightened inflammatory responses [62]. In our in vitro model, we show that AGS-associated microglia have increased cholesterol precursors, an accumulation of intracellular lipid droplets, and active morphologies that resemble inflammatory macrophages, suggesting that MVK deficiency may share characteristics with AGS pathology. Our clinical findings also show that a subset of AGS patients have abnormal lipid profiles, which could contribute to mechanisms that help explain the recent unintentional early screening of AGS by detection of elevated blood phospholipids [63].

Microglia are critical for healthy brain development [16, 41, 64], including synaptogenesis and circuit remodeling [19, 65]. How microglia affect oligodendrocyte development and myelination, however, is less well understood [66]. Studies have shown that microglia are responsible for the apoptosis and clearance of OPCs, engulfment of living OPCs, and phagocytosis of myelin membranes for the proper development of myelin [18, 54, 57]. Their contribution to white matter homeostasis has also been studied in the context of white matter diseases such as MS where they resemble a foamy-like morphology similar to the in vitro AGS phenotype we observed [26, 67]. Cholesterol metabolism in microglia and macrophages has been identified as a critical component of remyelination in experimental autoimmune encephalitis models of MS [25, 47, 50, 68]. We believe that the dysregulated cholesterol metabolism phenotype observed in our model of AGS-associated microglia could result in dysfunctional white matter homeostasis similar to the mechanism seen in MS.

Pathogenic mutations in microglial homeostasis genes, such as *TREM2, TYROBP*, and *CSF1R*, can cause leukodystrophies – rare genetic developmental disorders that affect white matter – suggesting that leukodystrophies may be good models for investigating microglial contribution to white matter development [69]. Loss-of-function mutations in *USP18* are associated with an IFN-mediated leukodystrophy, similar to AGS [70–72]. Knockout of *Usp18* in microglia induces microglia activation and migration to the white matter where structural integrity is compromised, and white matter disease persists [73]. Although the pathology of *USP18*-related leukodystrophy or the reason why microglia are selectively affected are unclear, we hypothesize that *USP18*-related leukodystrophy and AGS could share similar mechanisms.

The foundation of our understanding regarding brain development and dysfunction is mostly derived from mouse studies. Although useful, it is widely accepted that mice and humans have differences in brain development, specifically as it pertains to interactions between brain cells and microglia [74]. In studying AGS, human brain models are necessary since the *TREX1*-KO mouse model does not recapitulate the neurological symptoms of the disease [14]. Difficulties in obtaining fresh human brain tissue limit our understanding of this organ, but PSC technologies have allowed us to circumvent this by constructing 3D in vitro models of brain development [75]. In this study, we developed a 3D model where neurons, astrocytes, oligodendrocytes, and microglia are cultured together in an assembloid. Only a limited number of studies have included microglia in patterned neural organoid models [76–78]. Because of the importance of microglia in brain development, it is critical to include these cells in in vitro brain development models. Our study represents the first where both microglia and oligodendrocytes are cultured within the same organoid in the presence of neurons and astrocytes. Due to the nature of our assembloid model, we can better characterize the effects of genetically manipulated microglia on healthy brain development. In this study, we combined *TREX1*-KO microglia with control organoids and discovered that *TREX1*-KO microglia disrupts the oligodendrocyte lineage, suggesting that AGS leukodystrophy may be caused by microglia. Our model can be used to study other brain development disorders where microglia are affected such as leukodystrophies and interferonopathies.

Our scRNA-seq analysis uncovers an oligodendrocyte state bridging pre-OPCs with OPCs that we have named as t-OPC and that is characterized by the *OLIG1*^*+*^*PCDH15*^*+*^*PDGFRA*^*-*^ signature. Pre-OPCs are high *EGFR*-expressing gliogenic intermediate progenitor cells (IPCs) originated from radial glia [79], while t-OPC are low *EGFR*-expressing and high *DLL3-*expressing cells that have acquired only part of the OPC identity and that resemble primitive (pri)OPCs [80] (Fig. 4h). Both oligodendrocyte precursors express *EGFR*, and EGFR signaling is critical for terminal oligodendrogenesis [81]. In search for a potential EGFR ligand downregulated in *TREX1*-KO microglia that could explain the impaired pre-OPC/t-OPC to OPC transition, we observed that the most significantly downregulated gene by fold change in *TREX1*-KO microglia is *CHI3L1* (Supplemental Table 2). *CHI3L1* encodes a microglia-secreted glycoprotein, chitinase-3-like 1, reported to function as an EGFR ligand to induce oligodendrogenesis while reducing astrogliosis in human neural stem cells [82]. Moreover, CHI3L1 deficiency induces astrogliosis at expense of oligodendrogenesis [82], as we observed in *TREX1*-KO-microglia-supplemented organoids (Fig. 4d). We therefore propose that the CHI3L1-EGFR duo is a candidate ligand-receptor pair mediating *TREX1*-KO-microglia-mediated effects.

In summary, we identified a novel state of developmental microglia in AGS, which are L1 independent. We propose that AGS-associated microglia resemble WAMs and PAMs that are constitutively present during brain development which turn the otherwise protective phenotype into a destructive one. AGS-associated microglia have an amoeboid morphology and an increased rate of phagocytosis, which likely translates to aberrant phagocytosis of OPCs and a subsequent decrease in myelination. In addition, we speculate that the dysregulated cholesterol metabolism phenotype observed in AGS-associated microglia perturbs white matter homeostasis and development by a similar mechanism as seen in MS. We show that inhibiting HMG-CoA reductase using atorvastatin rescues the AGS microglial phenotypes to control conditions, a valuable finding that may be translated to other microglia-centric lipid-associated diseases such as MS and leukodystrophies. Finally, we developed a novel 3D brain development cellular model encompassing interactions between microglia, neurons, astrocytes, and oligodendrocytes, which can be used to study other neurodevelopmental diseases. This cellular model helped us uncover the novel connection between microglia and an intermediate stage in the oligodendrocyte lineage that we speculate might be mediated by a CHI3L1-EGFR ligand-receptor pair and is impaired in *TREX1*-associated AGS.

## Methods

### Study approval

All patient samples were obtained from individuals enrolled in a retrospective natural history study [Myelin Disorders Bioregistry at Children’s Hospital of Philadelphia (IRB#14-011236)]. Informed consent was obtained from all subjects.

### Cohort identification

Inclusion criteria were known AGS-related genotypes and availability of cholesterol measurements. Individuals without a known genotype or with absence of medical records were excluded.

### Lipid laboratory value collection and classifications

The following variables were collected for all samples as available: age at sample collection, genotype, and cholesterol panel results. Laboratory values were extracted from electronic medical records. Upper and lower limits of normal were collected as well. When patients were enrolled in a Janus kinase inhibitor study, only baseline values were collected. When multiple values were available, the first available level was provided.

### Maintenance of PSC cultures

Isogenic mutagenized H9 ESCs have been characterized previously [15]. Pluripotent stem cell colonies were cultured and passaged manually as small colonies on Matrigel-coated dishes (BD Biosciences, San Jose, CA, USA) with mTeSR™ Plus medium (StemCell Technologies, Vancouver, Canada).

### Mycoplasma testing

All cellular and tissue cultures were routinely tested for mycoplasma. Antibiotic-free media supernatants were collected, centrifuged, and resuspended in saline buffer. Ten microliters of each sample were assayed in a PCR with the following primers:

Forward: GGCGAATGGGTGAGTAAC;

Reverse: CGGATAACGCTTGCGACCT.

Mycoplasma-positive samples were immediately discarded and not used in the study.

### Differentiation of PSCs to microglia

A protocol previously developed [39] and adapted from [83] and [84] was used to differentiate PSCs to microglia. Prior to initiating differentiation, PSC colonies were gently dissociated using a 1:1 solution of Accutase^Ⓡ^ (Innovative Cell Technologies, San Diego, CA, USA) and PBS for 20 min at 37 °C and seeded at a concentration of about 800,000 cells per 10 cm matrigel-coated dish. Colonies were maintained in mTeSR™ Plus medium for about three days until the dish was about 70% confluent. The differentiation protocol consists of four sequential steps: primitive streak cell induction, hemangioblast-like hematopoietic precursors, myeloid differentiation, and monocyte generation. In the first step, primitive streak cells were induced by the addition of Bone Morphogenetic Protein - 4 (BMP-4, 80 ng/mL, Peprotech) to the mTeSR™ Plus medium with daily media changes for three days. In the second step (Day 4), cells were pushed to differentiate into hemangioblast-like hematopoietic precursors with the addition of a cocktail of three factors [Vascular Endothelial Growth Factor 121 (VEGF, 80 ng/mL, Peprotech), Stem Cell Factor (SCF, 100 ng/mL, Gemini Bio), and Fibroblast Growth Factor basic (bFGF, 25 ng/mL, Life Technologies)] in Microglial Precursor Differentiation Media (MPDM) which consists of StemPro-34 serum-free medium (SFM) (Thermo Fisher Scientific) supplemented with GlutaMax (2 mM, Thermo Fisher Scientific). In the third step (Day 6), the hematopoietic precursor cells were pushed towards myeloid differentiation with a different cocktail of factors [Fms-Like Tyrosine Kinase-3 ligand (FLT-3 ligand, 50 ng/mL, Gemini Bio), Interleukin-3 (IL-3, 50 ng/mL, Gemini Bio), Stem Cell Factor (SCF, 50 ng/mL, Gemini Bio), Thrombopoietin (TPO, 5 ng/mL, Peprotech), and Macrophage Colony-Stimulating Factor (M-CSF, 50 ng/mL, Gemini Bio)] in the MPDM for twelve days with media changes on days 6 and 10. In step four (Day 13), cells were fated into the monocytic lineage with supplementation of a cocktail of factors [FLT-3 ligand (50 ng/mL, Gemini Bio), M-CSF (50 ng/mL, Gemini Bio), and Granulocyte Macrophage Colony-Stimulating Factor (GM-CSF, 25 ng/mL, Peprotech)] in the MPDM with media changed twice a week. Cells produced in suspension in step 4 were recovered by centrifugation, resuspended in brain organoid media supplemented with M-CSF (50 ng/mL, Gemini Bio) and Interleukin-34 (IL-34, 50 ng/mL, BioLegend), replated and allowed to mature for one week prior to use in experiments.

### RT-qPCR analysis

Total RNA was obtained from microglia using the RNeasy Plus Micro Kit (QIAGEN, Hilden, Germany). RNA was then used to generate cDNA using the QuantiTect^Ⓡ^ Reverse Transcription Kit (QIAGEN, Hilden, Germany), and 10 ng of cDNA was assayed in each 20 µL qPCR reaction in triplicate using individual primers (Integrated DNA Technologies, Coralville, IA, USA) and iQ™ SYBR^Ⓡ^ Green Supermix (Bio-Rad Laboratories, Hercules, CA, USA). Thermal cycling and plate readings were performed on a Bio-Rad CFX Connect™ Real-Time System. Relative gene expressions were calculated using the ΔΔC_T_ method and GAPDH as a reference gene. For the detection of L1 ORFs, 10 ng of microglia cDNA was assayed in 12 µL qPCR reactions using TaqMan™ probes and the TaqMan™ Fast Advanced Master Mix (Life Technologies). The L1 probes were described previously [15].

ORF1:ATGGGGAAAAAACAGAACAGAAAAACTGGAAACTCTAAAACGCAGAGCGCCTCTCCTCCTCCAAAGGAACGCAGTTCCTC

ORF2:GCTCATGGGTAGGAAGAATCAATATCGTGAAAATGGCCATACTGCCCAAGGTAATTTACAGATTCAATGCCATCCCCATC

ORF2-3′UTR:TGGAAACCATCATTCTCAGTAAACTATCGCAAGAACAAAAAACCAAACACCGCATATTCTCACTCATAGGTGGGAATTGA

List of Primers:

**Table:** 

Target	Forward sequence	Reverse sequence
*GAPDH*	GTCTCCTCTGACTTCAACAGCG	ACCACCCTGTTGCTGTAGCCAA
*ABCA1*	CAGGCTACTACCTGACCTTGGT	CTGCTCTGAGAAACACTGTCCTC
*ACAT1*	CCAGCCACTAAGCTTGGTTCCA	GTAGGAGCTTGTCCTTCACCTC
*ACAT2*	TGGTGCCTTAGCTGCTGTTCCT	GGCTTGTCTAACAGGATTCTGCC
*DHCR7*	TCCACAGCCATGTGACCAATGC	CGAAGTGGTCATGGCAGATGTC
*DHCR24*	CAGGAGAACCACTTCGTGGAAG	CACATGCTTAAAGAACCACGGC
*HMGCR*	GACGTGAACCTATGCTGGTCAG	GGTATCTGTTTCAGCCACTAAGG

### Cytokine assay

For type I IFN cytokine measurements in culture supernatants, 50,000 cells were seeded into individual wells of a 96-well plate, allowed to mature for 4–7 days, then treated and cultured for 48 h prior to collecting supernatant. Concentrations of cytokines and chemokines were quantified using the U-PLEX Interferon Combo (hu) kit (Meso Scale Discovery) according to the manufacturer’s instruction. Measurements were performed on the MSD Imager MESO QuickPlex SQ 120.

### Microglia zymosan phagocytosis assay

For phagocytosis assays, microglial precursor cells were seeded onto 96-well black-wall clear-bottom plates (Greiner) at a concentration of 35,000 cells in 200 μL of media per well. Cells were allowed one week to mature prior to addition of treatments. About 18–24 h after treatment, 5 µg of pHrodo Red Zymosan Bioparticles (Thermo Fisher Scientific) and Hoechst 33342, a cell-permeant live-cell nuclear stain, in 100 μL of media was added per well. Two phase contrast and red fluorescent images were acquired every hour for 4 h using an Incucyte S3 live-cell analysis system (Essen Bioscience) and fluorescence was measured at excitation wavelengths of 560 nm for pHrodo and 350 nm for Hoechst 33342 every hour for 4 h using an Infinite 200Pro microplate reader (Tecan). After 4 h, brightfield, red fluorescent, and DAPI images were taken and merged using an EVOS imaging system (Thermo Fisher Scientific). Uptake of pHrodo-zymosan particles was calculated by normalizing its fluorescence to the fluorescence of the nuclear stain each hour. Images were used for visual confirmation and representation.

### Microglia morphology analysis

To determine differences in morphology of PSC-derived microglia, we utilized the automated imaging and analysis software of the Incucyte S3 live-cell analysis system (Essen Bioscience). Immediately prior to the addition of pHrodo Red Zymosan Bioparticles (Thermo Fisher Scientific) to measure phagocytosis and after cells were treated for at least 18 h, three brightfield images were taken per well at 20X magnification. For analysis, the NeuroTrack module was modified to accommodate our PSC-derived microglia with a minimum cell width of 15 µm and a minimum cell-body cluster of 100 µm^2^. Process length and number of branch points were normalized to the cell-body cluster area.

### Microglia immunocytochemistry

Microglial precursor cells were seeded onto 8-chamber glass slides at a density of 50,000 cells per chamber. After one week of maturation, cells were fixed with 4% paraformaldehyde for 15 min at room temperature and washed with phosphate-buffered saline (PBS). Cells were blocked for 1 h in blocking solution [10% Bovine Serum Albumin (BSA)/1% Triton X-100/1$\times$ PBS]. Incubation with primary antibodies was performed in the same blocking solution overnight at 4 °C. Primary antibodies used were rabbit anti-IBA1 (Wako; 019-19741; 1:500), goat anti-TREM2 (R&D Systems; AF1828SP; 1:500), rabbit anti-CX3CR1 (Bio-Rad; AHP1589; 1:500), and rabbit anti-P2Y12R (Alomone; APR-020; 1:500). After 3 washes in 1$\times$ PBS, cells were labeled with fluorescently labeled secondary antibodies for 2 h, and nuclei were counterstained with 1 µg/mL DAPI (Thermo Fisher Scientific) for 10 min. Slides were mounted with ProLong Gold anti-fading solution (Thermo Fisher Scientific). Images were taken using a Zeiss fluorescence microscope equipped with Apotome (Axio Observer Apotome, Zeiss).

### Microglia flow cytometry

Microglia were harvested, counted, then washed twice with 1$\times$ PBS/1% FBS at 300 $\times$ *g* for 5 min. Conjugated antibodies were added in the cell mixture and incubated at room temperature in the dark for 20–30 min. Antibodies used include the following: CD45 BV786 and CD11b APC, both from BD Biosciences. Cells were then washed twice in the same buffer as previously and resuspended in 500 µL buffer with the viability dye, 7-AAD (BD Biosciences). Cells were analyzed using a FACSCanto II (BD Biosciences) flow cytometer and FlowJo software.

### Bulk RNA sequencing

Total RNA was obtained from microglia using the RNeasy Plus Micro Kit (QIAGEN, Hilden, Germany). Total RNA was assessed for quality using an Agilent Tapestation 4200, and samples with an RNA Integrity Number (RIN) greater than 8.0 were used to generate RNA sequencing libraries using the Illumina® Stranded mRNA Prep (Illumina, San Diego, CA). Samples were processed following manufacturer’s instructions. Resulting libraries were multiplexed and sequenced with 100 basepair (bp) Paired End reads (PE100) to a depth of approximately 25 million reads per sample on an Illumina NovaSeq 6000. Samples were demultiplexed using bcl2fastq Conversion Software (Illumina, San Diego, CA).

Quality of fastq files was assessed using FASTQC (version 0.11.8). Adapters were trimmed using cutadapt (version 1.16). Reads were mapped to the reference human genome (hg38) using STAR (version 2.7.8a). Gene counts were generated using featureCounts (version 2.0.1). Differential expression analysis was performed using DESeq2 (version 1.22.1) [85] with a two-factor design comparing control versus *TREX1*-KO microglia and accounting for the cell line (design formula: ~Line + Genotype). To aggregate transcript abundances into gene-level counts, we used tximport (version 1.10.0) [86]. Genes with an effect size (Log2FC) > |1.5| were filtered, and DE genes were determined based on their *p*-adj values (<0.05) calculated using the Benjamini–Hochberg multiple test correction.

To visualize DE genes between control and *TREX1*-KO microglia, a volcano plot was generated using ggplot2 (version 3.1.0) with a significance threshold of 0.05. A heatmap was also generated using gplots (version 3.0.1) where data was log2-transformed and rows were clustered using the Euclidean distance method. Gene ontology (GO) analysis was performed using goseq (version 1.44.0) [87] with parameters set for the Wallenius method and Benjamini–Hochberg multiple test correction.

### Total sterol/oxysterol analysis

Lipid analysis was performed at the UCSD Lipidomics core [88]. Microglial cell pellets consisting of 2 million cells each were homogenized into 250uL of 10% methanol in water. An internal standard mix of 25-Hydroxycholesterol-d6, Desmosterol-d6, and Campesterol-d6 (Avanti Polar Lipids) was added to 100uL of the homogenate. Samples were saponified for 1.5 h at 37 °C with a final concentration of 0.2 N KOH. Samples were extracted with 500uL of butanol/methanol (3:1, v/v), heptane/ethyl acetate (3:1, v/v), and 1% acetic acid In water [89]. Extracts were brought to dryness and taken up in 90% methanol in water and run on a Waters Acquity UPLC interfaced with an AB Sciex 6500 QTrap mass spectrometer equipped with an APCI probe. Source settings were: Curtain Gas = 20, Collision Gas = Medium, Ion Spray Voltage = 5500, Temperature = 400, GS1 = 25, NC = 1. A Phenomenex Kinetex C18 1.7 μM 2.1 mm × 150 mm column was used for chromatographic separation. A 30 min step gradient was employed using 70/30 acetonitrile/water with 5 mM ammonium acetate as Buffer A and 50/50 acetonitrile/water with 5 mM ammonium acetate as Buffer B with a flow of 0.5 mL/min. The gradient started at 0%B for 2 min, ramped to 10%B over 4 min, 15%B over 9 min, 50%B over 11 min, 100%B over 2 min, then held at 100%B for 2 min. Sterol species were identified by mass spectrometry using 30 MRMs (Multiple Reaction Monitoring) in positive mode. Standard curves were obtained in parallel using identical conditions. Data analysis was performed with Analyst and Mulitquant software packages.

### Lipid-droplet assay

To measure lipid droplets, microglial precursor cells were plated onto 8-chamber glass slides at a density of 50,000 cells per chamber. Cells were allowed to mature for 4 days prior to the addition of treatments. About 18–24 h after treatment, cells were incubated with fresh media supplemented with BODIPY™ 493/503 (Thermo Fisher Scientific) and Hoechst 33342 for 30 min. Cells were then washed with PBS and fixed in 4% Paraformaldehyde for 15 min at room temperature. Slides were then mounted using Prolong Gold and allowed to dry overnight prior to imaging.

Lipid droplets were imaged at 63× magnification using a Zeiss fluorescence microscope equipped with Apotome (Axio Observer Apotome, Zeiss). Lipid droplets were counted and their area was measured using the Fiji image processing package equipped with ImageJ2 [90].

### Microglia drug treatment

Control cells were treated with either DMSO or polyinosinic–polycytidylic acid sodium salt [poly (I:C), 50 μg/mL, Sigma–Aldrich], and *TREX1*-KO cells were treated with either DMSO, Atorvastatin (1 µM, Sigma–Aldrich), or RTis [3TC (10 µM, Sigma–Aldrich) and D4T (1 µM, Sigma–Aldrich)].

### Differentiation of PSCs to oligodendrocyte-containing brain organoids and assembloid generation

For the generation of oligodendrocyte-containing organoids (hOLS or organoids), we used a previously published protocol [37] with some modifications. Briefly, PSC colonies were dissociated using Accutase (Thermo Fisher Scientific; diluted with an equal volume of 1$\times$ PBS) for 20 min at 37 °C. After centrifugation for 3 min at 150 $\times$ *g*, the individualized cells were resuspended in mTeSR1 Plus medium (Stem Cell Technologies) supplemented with 10 µM Rho kinase inhibitor (Y-27632; Calbiochem, Sigma–Aldrich), and 3 $\times$ 10^6^ cells were added per AggreWell-800 (Stem Cell Technologies) well and centrifuged to capture cells in microwells. Spheroids consisting of about 10,000 cells were collected from the microwells after 24 h by pipetting up and down with a cut pipet tip and transferred to an ultra-low attachment 6-well plate. Neural differentiation was induced by supplementation with 2.5 µM dorsomorphin (R&D Systems) and 10 µM SB431542 (Stemgent) in mTeSR1 Plus medium for 5 days. Organoids were maintained on an orbital shaker inside a CO_2_ incubator at 95 rpm. On day 4, 5 µM of the Wnt inhibitor IWP-2 (Selleck Chemical) was supplemented until day 24. On day 6, mTeSR1 Plus medium was replaced with differentiation and maintenance medium (DMM) consisting of DMEM/F12 (Thermo Fisher Scientific) containing B27 without Vitamin A (Thermo Fisher Scientific), N2 NeuroPlex supplement (Gemini Bio-Products), non-essential amino acids (Thermo Fisher Scientific), penicillin/streptomycin (Thermo Fisher Scientific), GlutaMAX (Thermo Fisher Scientific), and 0.1 mM β-mercaptoethanol (Thermo Fisher Scientific). Media was supplemented with the following growth factors until day 24: 20 ng/mL EGF (Peprotech) and 20 ng/mL bFGF (Thermo Fisher Scientific). On day 12 until day 24, 1 µM smoothened agonist SAG (Sigma–Aldrich) was added to the media. On day 25 until day 36, DMM was supplemented with the following cocktail of factors: 25 µg/mL insulin (Sigma–Aldrich), 60 ng/mL T3 (Sigma–Aldrich), 100 ng/mL biotin (Sigma–Aldrich), 20 ng/mL NT-3 (Peprotech), 20 ng/mL BDNF (Peprotech), 1 µM cAMP (Thermo Fisher Scientific), 5 ng/mL hepatocyte growth factor (Peprotech), 10 ng/mL IGF-1 (Peprotech), and 10 ng/mL PDGF-AA (R&D Systems). On day 37 until endpoint, DMM was supplemented with the following cocktail of factors: 25 µg/mL insulin (Sigma–Aldrich), 60 ng/mL T3 (Sigma–Aldrich), 100 ng/mL biotin (Sigma–Aldrich), 1 µM cAMP (Thermo Fisher Scientific), and 20 µg/mL ascorbic acid (Sigma–Aldrich).

On Day 37, organoids were prepared for co-culture with microglia for assembloid generation. Individual organoids were placed into wells of ultra-low attachment u-bottom 96-well plates. Microglia precursor cells were resuspended in DMM supplemented with the day 37 organoid cocktail of supplements plus 50 ng/mL M-CSF (Gemini) and 50 ng/mL IL-34 (Biolegend) and 100,000 cells were added per well of the 96-well plate on top of the organoid to a total volume of 200 µL of media. Organoids and microglia were kept in the 96-well plate for 1 week prior to transferring to a 6-well plate and continuing to culture under orbital rotation.

### Assembloid dissociation for single-cell RNA sequencing

Single-cell RNA-seq was performed on dissociated assembloids composed of control organoids and control or *TREX1*-KO microglia at 3 months in vitro, totaling 5 libraries consisting of 3 replicate libraries of assembloids composed of control organoids and control microglia, and 2 replicate libraries of assembloids composed of control organoids and *TREX1*-KO microglia. For each library, a total of 4–5 assembloids were used.

Assembloids were dissociated to a single-cell suspension via a combination of mechanical dissociation and enzymatic digestion. First, assembloids were mechanically broken apart using pipette tips in a 6-well plate. Immediately after, a combination of Accutase (Thermo Fisher Scientific) and Papain supplemented with DNase (Worthington) were added to the plate and incubated under orbital rotation. Clumps were dissociated every 10 min by pipetting up and down for a total of 30 min. Dissociated cells were pelleted (3 min, 100 $\times$ *g*) and resuspended in PBS. Cells were counted and viability was checked using an automated cell counter (Bio-Rad).

### AmpliDrop 3′ scRNA-seq analysis

Single-cell suspensions were processed to generate single-cell libraries using AmpliDrop^TM^ technology according to the manufacturer’s instructions (preprint in preparation) and services provided by UST (Universal Sequencing Technology Corp., Carlsbad, CA). Approximately 9000 to 14,000 live cells were processed per sample. Fixed cells were permeabilized, reverse transcribed, and tagmented. Treated cells were encapsulated individually in water-in-oil droplets generated by controlled pipetting with an electronic pipette in a PCR tube. Tagged cDNA in a cell was subsequently amplified in the droplet along with unique barcode tags in a MiniAmp Thermal Cycler (Thermo Fisher), resulting in amplified 3′ end of cDNA fragments attached with a cell-specific barcode. Illumina sequencing adapters were added to barcoded cDNA to generate final single-cell libraries. Cleanup and size selection was performed using HighPrep PCR Clean-up MagBio magnetic beads (MagBio Genomics). Library quantification and sizing were made using High Sensitivity D1000 Screen Tape and the 4150 TapeStation system (Agilent Technologies). Samples were sequenced on the NextSeq instrument (Illumina) as single-end reads (10,500–17,500 mean reads per cell).

### AmpliDrop 3′ scRNA-seq data processing, exploration, and visualization

Fastq files generated from AmpliDrop^TM^ libraries were processed using AmpliDrop^TM^ Analysis Pipeline v1.0 (UST). Processed fastq files were then used as input for Cell Ranger v5.0.1 (10x Genomics). Single Cell 3′ v3 (--chemistry threeprime) was the selected chemistry with introns (--include-introns) in Cell Ranger. Sequencing reads were aligned with STAR (v2.7.6) using the GRCh38 human reference genome. The R packages Seurat (v4.1.1) and Harmony (v0.1.0) were used for data filtering, normalization, scaling, dimensionality reduction, clustering, expression analysis, exploration, and visualization (DotPlot). Loupe Browser (v6.1.0) was used for data exploration and visualization (UMAP plots using Seurat-generated projections). Microsoft Excel (v16.65) was used for visualization (staggered and proportional plots).

Seurat objects were individually created from each sample using the CreateSeuratObject() function (min.cells = 3 and min.features = 200). Cells that had fewer or greater than 200 and 3000 features, respectively, and contained greater than 5% of reads from mitochondrial genes were considered low quality and removed from further analysis. No software (such as DoubletFinder) was used to infer and remove doublets owing to the risk of accidentally removing transitioning cell substates. Instead, we manually examined the data for unexpected co-localization of well-known cell-type-specific gene markers.

Data were normalized and scaled using the NormalizeData() and ScaleData() functions, respectively (scale factor = 10,000). Data were integrated based on 2000 highly variable genes using the FindVariableFeatures() function (selection.method = vst). Anchors between individual datasets were identified based on the subset of highly variable genes using the FindIntegrationAnchors() function (dims = 1:20). Anchors were then inputted into the IntegrateData() function (dims = 1:20) to create a batch-corrected expression matrix of all cells. Principal component analysis and UMAP dimension reduction were performed using the RunPCA() and RunUMAP() functions (npcs = 30; dims = 1:20). A nearest-neighbor graph was then calculated using the FindNeighbors() function (dims = 1:20), followed by clustering using the FindClusters() function (resolution = 0.5).

Cellular identity was determined by finding differentially expressed genes for each cluster using the FindMarkers() function and comparing the identified markers to known cell-type-specific genes, listed in Fig. 4c. Seurat-defined clusters were re-named based on these gene markers. Due to the relatively small fraction of microglial cells, microglia were identified in the context of a larger single-cell dataset characterized by *C1QA*, *CX3CR1*, *CCL3*, *CD74*, and *C1QB* co-expression.

The DotPlot() function was used to visualize the expression of selected genes in the oligodendrocyte subpopulations. Gene expression on UMAP plots was generated with Loupe Browser and Seurat/Harmony-corrected projections (Fig. S4A). Gene expression on UMAP plots visualized as densities was generated with the Nebulosa (v.1.8.0) package (Fig. 4b, f). Seurat/Harmony projections were exported using the cbind() function and uploaded directly into the Loupe Browser. Cell proportions were calculated with Excel from Seurat object labeling.

Pseudotime analysis was performed by sub-setting the Pre-OPC, T-OPC, OPC, Dividing OPC, and Astroglia populations from the Seurat object and creating a CellDataSet object compatible with the Monocle3 (v.1.3.4) package. Cell identities were transferred from Seurat object to a CellDataSet object using the AddMetaData() function. After multi-dimensional reduction using the reduce_dimention() function, we manually determined the start of the pseudotime (time zero) on the Pre-OPC population prompted by the order_cells() function.

### Immunohistochemistry of assembloids

After 3 months of culture, assembloids were fixed with 4% paraformaldehyde overnight at 4 °C and cryoprotected in 30% sucrose for at least 24 h. Assmebloids were then embedded in TissueTek (Leica Microsystems) and sectioned on a Leica CM1850 cryostat to produce 20 µm sections. For staining, slides were air-dried for 10 min, blocked with 0.1% Triton X-100/3% BSA/1$\times$ PBS for 1 h at room temperature, and incubated with primary antibodies in the blocking solution overnight at 4 °C. For myelin visualization, the primary antibody used was rat anti-MBP (Millipore Sigma; MAB386; 1:500). After incubation in a solution containing the primary antibody, slides were washed three times in 1$\times$ PBS and incubated with fluorescently labeled secondary antibodies (Alexa Fluor 488- or 555-conjugates antibodies; 1:1000; Thermo Fisher Scientific) in blocking solution for 2 h at room temperature. After further washes in 1$\times$ PBS, slides were counterstained with DAPI solution (1 µg/mL) for 10 min and mounted with ProLong Gold anti-fading solution (Thermo Fisher Scientific). All images were taken using a Zeiss fluorescence microscope equipped with Apotome (Axio Observer Apotome, Zeiss).

### Electron microscopy

Electron microscopy was performed at the CMM Electron Microscopy Facility at the University of California San Diego. 150-day-old assembloids were fixed with 2%glut. in 0.10 M cacodylate buffer. and further postfixed in 1% OsO_4_ in 0.1 M cacodylate buffer for 1 h on ice. The cells were stained all at once with 2% uranyl acetate for 1 hr on ice, following which they were dehydrated in graded series of ethanol (50–100%) while remaining on ice. The cells were then subjected to 1 wash with 100% ethanol and 2 washes with acetone (10 min each) and embedded with Durcupan. Sections were cut at 60 nm on a Leica UCT ultramicrotome, and picked up on 300 mesh copper grids. Sections were post-stained with 2% uranyl acetate for 5 min and Sato’s lead stain for 1 min. Grids were analyzed using a JEOL 1400 plus (JEOL, Peabody, MA, USA) transmission electron microscope equipped with a bottom-mount Gatan OneView (4k × 4k) camera (Gatan, Pleasanton, CA, USA).

### Statistical analysis

Statistical analyses were performed using GraphPad Prism. The reported values are means ± SD or SEM, noted in the figure legend for each panel. Unless otherwise noted in the figure legend, *n* = 2 cell lines for genotypes CTRL (WT63, WT83) and *TREX1*-KO (V63fs, E83fs). Statistical significance was determined using Student’s t tests when comparing two groups or ordinary one-way ANOVA when comparing three or more groups. Variance within datasets for each experiment were similar between the CTRL and *TREX1*-KO groups being compared.

### Supplementary information

Supplemental Figures and Tables Legends

Supplemental Figure 1

Supplemental Figure 2

Supplemental Figure 3

Supplemental Figure 4

Supplemental Table 1

Supplemental Table 2

## Data Availability

Data supporting the findings in this study are included within the Supplementary Material. RNA sequencing and single-cell RNA sequencing raw and processed data generated during this study were deposited at the Gene Expression Omnibus (GEO) of the National Center for Biotechnology Information (NCBI) under accession numbers GSE216702 and GSE216718, which are available publicly without restriction. All data and/or analyses generated during the study are available from the corresponding author upon reasonable request.
