# Supplementary figures and images for: TREX1 is required for microglial cholesterol homeostasis and oligodendrocyte terminal differentiation in human neural assembloids
Source: Mol Psychiatry. Author manuscript; Available in PMC 2024 Jun 6. (PMC11153041; doi:10.1038/s41380-023-02348-w)

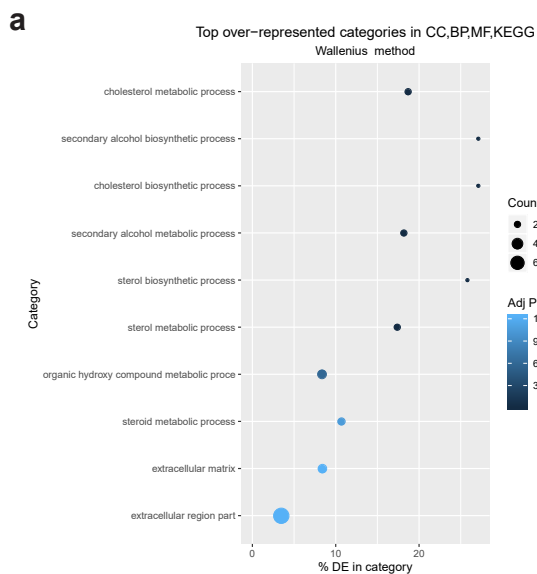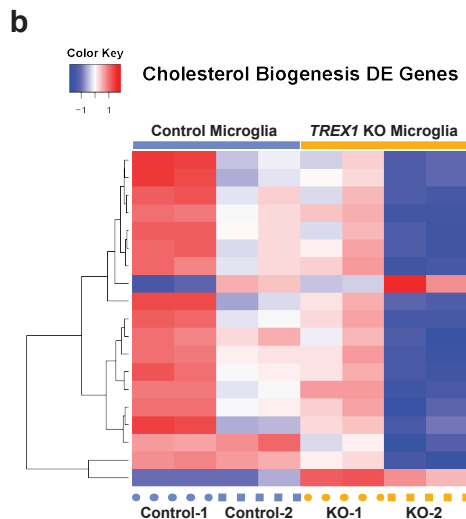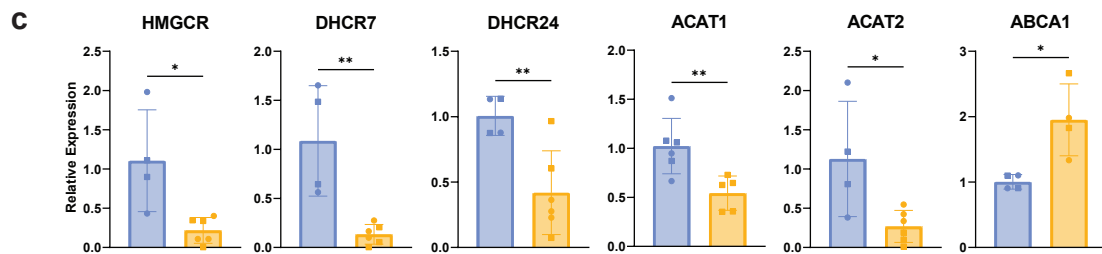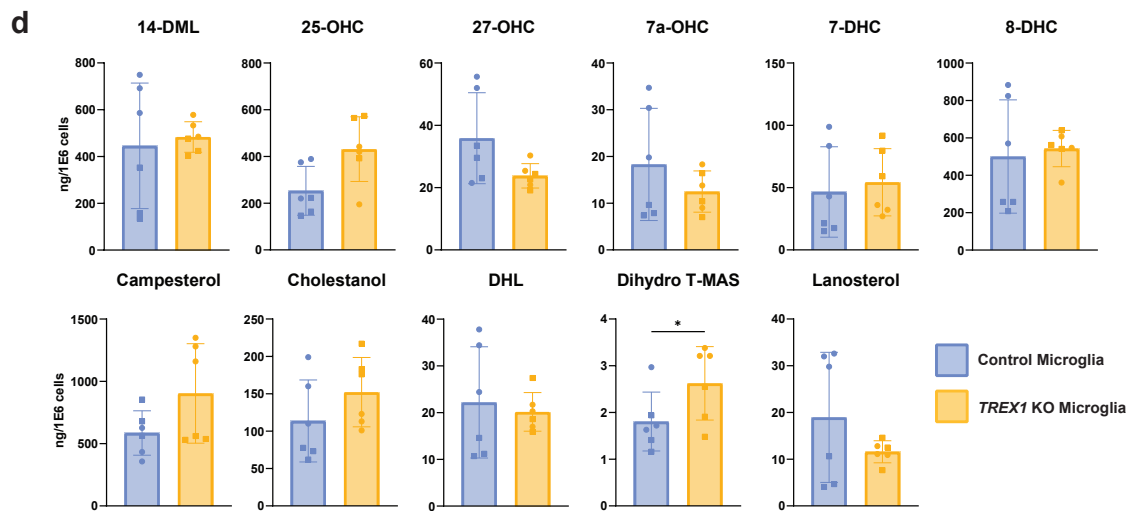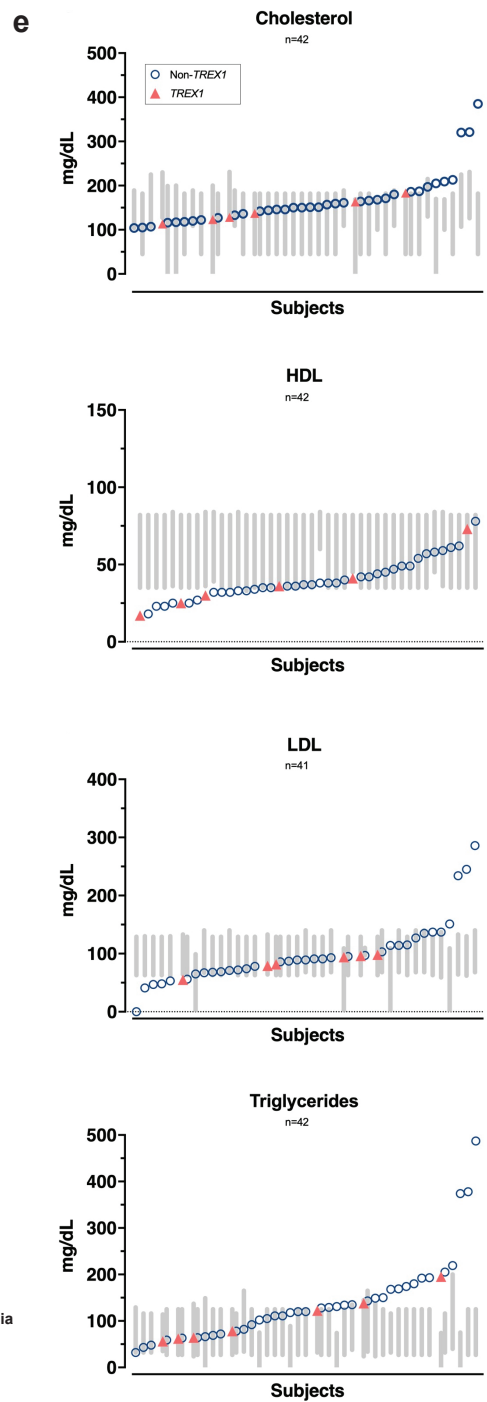

Supplement: Supp Material 5 [file NIHMS1981023-supplement-Supp_Material_5.pdf]

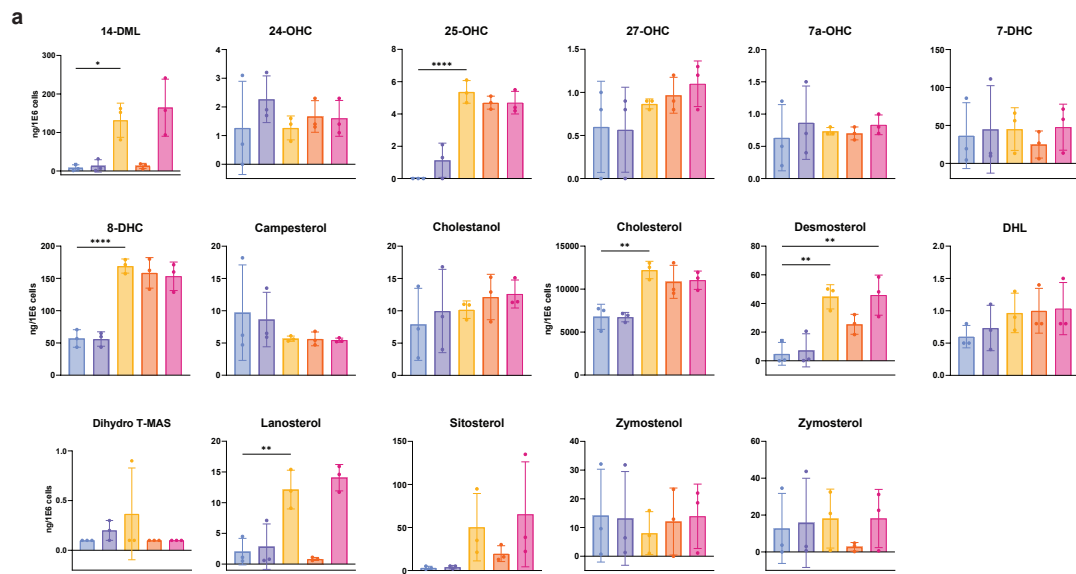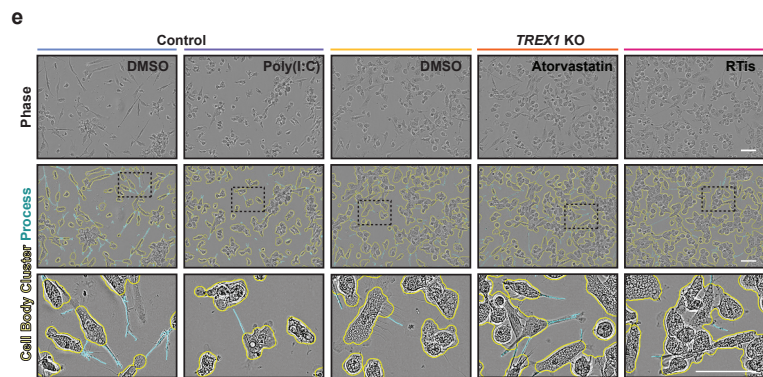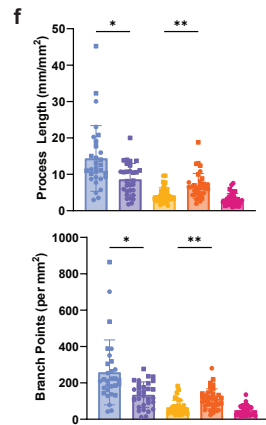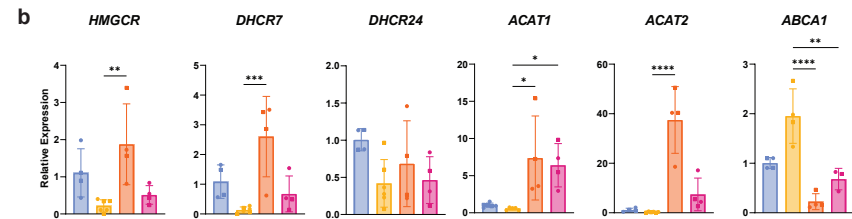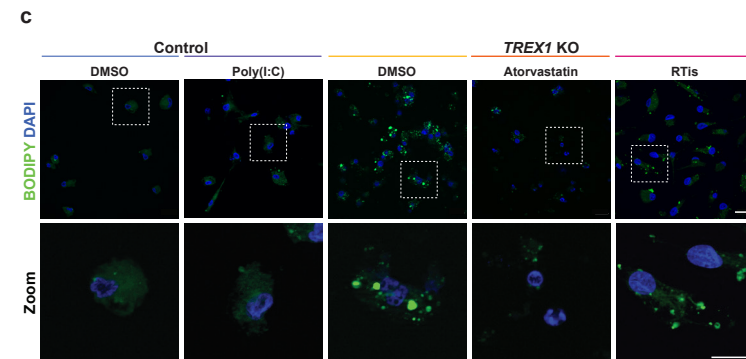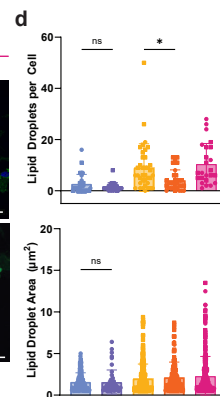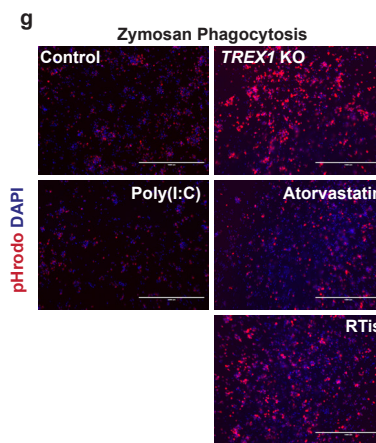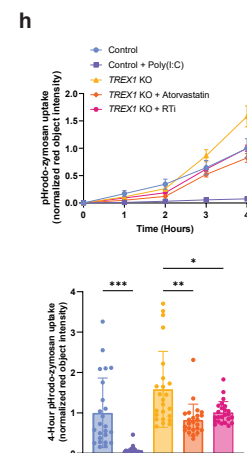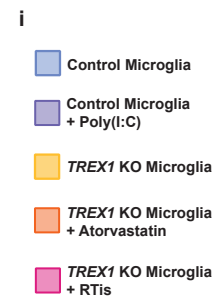

Supplement: Supp Material 4 [file NIHMS1981023-supplement-Supp_Material_4.pdf]

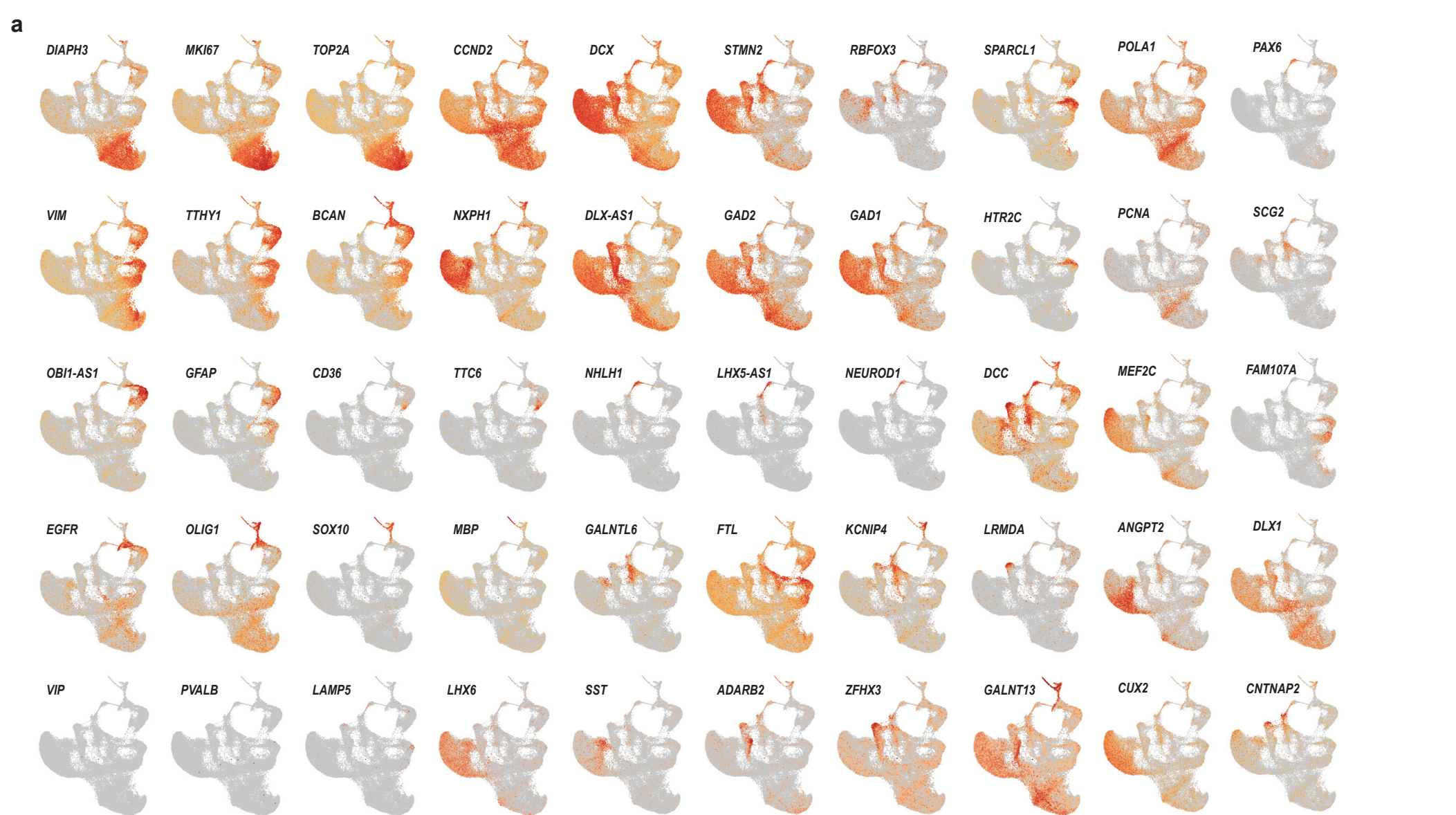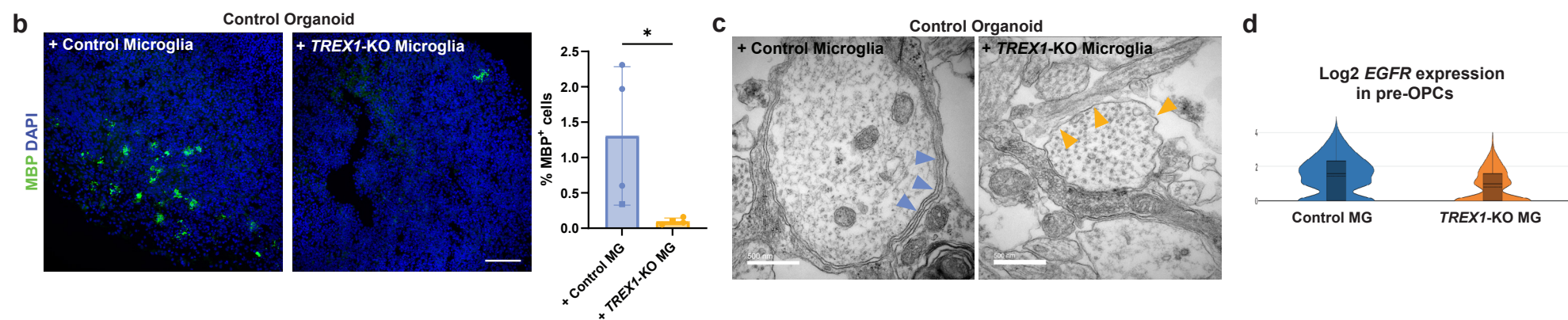

Supplement: Supp Material 3 [file NIHMS1981023-supplement-Supp_Material_3.pdf]

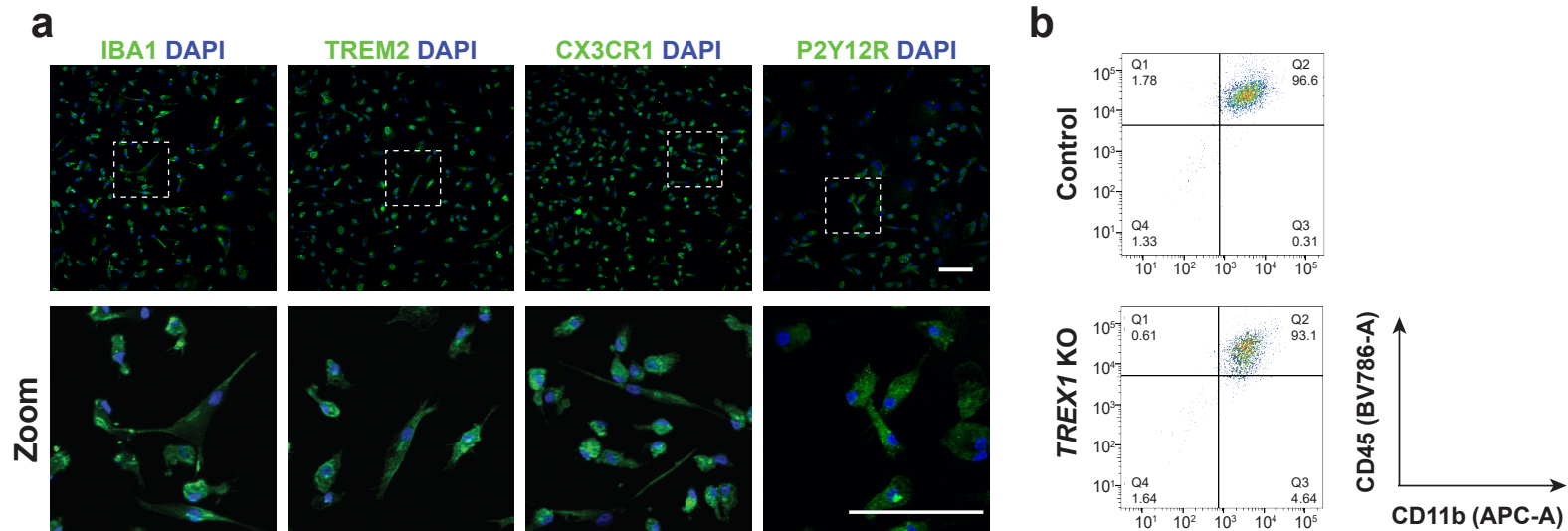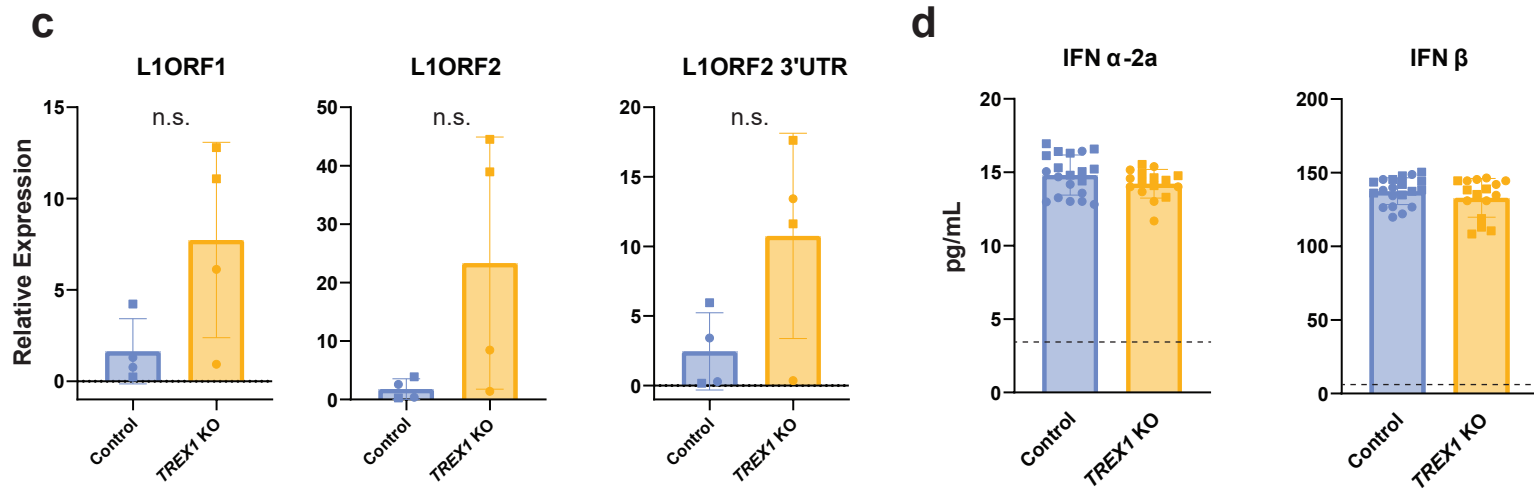

Supplement: Supp Material 6 [file NIHMS1981023-supplement-Supp_Material_6.pdf]
