# Supplementary material for: TREX1 is required for microglial cholesterol homeostasis and oligodendrocyte terminal differentiation in human neural assembloids
Source: Mol Psychiatry. Author manuscript; Available in PMC 2024 Jun 6. (PMC11153041; doi:10.1038/s41380-023-02348-w)
Supplement: Supp Material 7 [file NIHMS1981023-supplement-Supp_Material_7.docx]

**SUPPLEMENTAL INFORMATION**

**Fig. S1: Related to Fig. 1 *TREX1*-KO microglia demonstrate an active phenotype.**

**a** Representative fluorescent images of microglia-like cells showing expression of canonical microglial markers such as IBA1, TREM2, CX3CR1, and P2Y12R. Scale bar is 100µm. **b** Flow cytometry of microglia-like cells showing double-positive staining of CD45 (BV786) and CD11b (APC). **c** Relative expression (RT-qPCR) of L1 in microglia-like cells. Expression was quantified with three different primers corresponding to different regions of L1. The presented values are means ± SEM (Control, n = 4 independent microglia differentiation experiments; *TREX1*-KO, n = 3 independent microglia differentiation experiments). **d** Concentration (pg/mL) of IFN-α and IFN-β in 48-hour microglia conditioned media. Dotted line represents lower limit of detection. The presented values are means ± SEM (n = 4 wells per cell line). Symbols in bar graphs indicate cell lines used: circles, Control-1 and *TREX1*-KO-1 pair; squares, Control-2 and *TREX1*-KO-2 pair. Colors in graphs represent either the control lines (blue) or the *TREX1*-KO lines (yellow).

**Fig. S2: Related to Fig. 2 Cholesterol synthesis is dysregulated in *TREX1*-KO microglia**

**a** Top over-represented GO terms plot for RNAseq of control versus *TREX1*-KO microglia showing cholesterol metabolic process is the top over-represented category. CC = cellular component; BP = biological process; MF = molecular function; KEGG = Kyoto Encyclopedia of Genes and Genomes. **b** Heatmap of the top differentially expressed genes between control and *TREX1*-KO microglia that relate to cholesterol biogenesis. Red are upregulated genes and blue are downregulated genes. Rows are clustered using the Euclidean distance method. **c** Relative expression (RT-qPCR) of cholesterol synthesis genes (*HMGCR*, *DHCR7*, *DHCR24*, *ACAT1*, *ACAT2*, and *ABCA1*) in control and *TREX1*-KO microglia. The presented values are means ± SEM (n = 8; 2 independent experiments and 4 cell lines). **d** Total sterol/oxysterol analysis of control and *TREX1*-KO microglia. The presented values are means ± SEM (n = 12; 3 independent microglia differentiation experiments and 4 lines). **e** Lipid profiles of patients with AGS. Lipid profiles included total cholesterol (n=42), high density lipoprotein (HDL; n=42), low density lipoprotein (LDL; n=41), and triglycerides (n=44). The upper-lower bounds are shown by the grey bars. Each circle represents values from non-*TREX1*-related AGS subjects; closed triangles represent values obtained from *TREX1* AGS subjects. Symbols in bar graphs indicate cell lines used: circles, Control-1 and *TREX1*-KO-1 pair; squares, Control-2 and *TREX1*-KO-2 pair. Colors in graphs represent either the control lines (blue) or the *TREX1*-KO lines (yellow). *p < 0.05, **p < 0.01.

**Fig. S3: Related to Fig. 3 Atorvastatin rescues cholesterol-associated and active *TREX1*-KO microglia phenotype**

**a** Total sterol/oxysterol analysis of control and *TREX1*-KO microglia treated with DMSO, control microglia treated with poly(I:C), and *TREX1*-KO microglia treated with atorvastatin or RTis. The presented values are means ± SEM (n = 12; 3 independent microglia differentiation experiments and 4 lines). **b** Relative expression (RT-qPCR) of cholesterol synthesis genes (*HMGCR*, *DHCR7*, *DHCR24*, *ACAT1*, *ACAT2*, and *ABCA1*) in control and *TREX1*-KO microglia treated with DMSO and *TREX1*-KO microglia treated with either atorvastatin or RTis. The presented values are means ± SEM (n = 16; 2 independent experiments and 4 cell lines). **c** Representative images of BODIPY-stained treated microglia. Scale bars are 20µm.  **d** Top: Quantification of number of lipid droplets per cell. Bottom: Quantification of lipid droplet area. The presented values are means ± SEM (n = 30 cells per condition). **e** Representative brightfield images of treated microglia showing an overlay of outlines depicting cell body clusters and cell processes determined using the Incucyte S3 live cell analysis system. Scale bar is 100µm. **f** Top: Quantification of process length to analyze microglial morphology. Bottom: Quantification of number of branch points. The presented values are means ± SEM (n = 30 wells per condition). **g** Representative images of treated microglia 4 hours post pHrodo-zymosan phagocytosis assay. Scale bars are 1000µm.  **h** Top: Line graph of pHrodo-zymosan uptake over 4 hours. Bottom: Four hour pHrodo-zymosan uptake normalized fluorescence intensity. The presented values are means ± SEM (n = 24 wells per condition). Symbols in bar graphs indicate cell lines used: circles, Control-1 and *TREX1*-KO-1 pair; squares, Control-2 and *TREX1*-KO-2 pair. Colors in graphs represent the control lines (blue), control lines treated with poly (I:C) (purple), *TREX1*-KO lines (yellow), *TREX1*-KO lines treated with atorvastatin (orange), or *TREX1*-KO lines treated with RTis (magenta). *p < 0.05, **p < 0.01, ***p < 0.001, ****p < 0.0001.

**Fig. S4: Related to Fig. 4 TREX1 in microglia is necessary for development of myelinating oligodendrocytes**

**a** UMAP plots of combined scRNA-Seq libraries showing individual expression of selected marker genes used for assigning subpopulations of cells. Each dot represents a cell. The darker the color, the higher the expression of the gene relative to all other cells. **b** Left: Representative images of assembloids composed of control organoids co-cultured with either control or *TREX1*-KO microglia showing increased expression of myelin basic protein (MBP) in assembloids containing control microglia. Scale bar is 100µm. Right: Quantification of percent MBP-positive cells in images. The presented values are means ± SEM (assembloids containing control MG: n = 4; assembloids containing *TREX1*-KO MG: n = 4). **c** Representative transmission electron microscopy images of control assembloids containing either control or *TREX1*-KO microglia showing early myelination ultrastructures in the samples containing control microglia but not in the samples containing *TREX1*-KO microglia. Scale bars are 500nm. Triangles point at early myelination structures. **d** Violin plots representing the log2 *EGFR* expression in the pre-OPC cluster between assembloids containing control microglia and *TREX1-*KO microglia. MG: microglia. Symbols in bar graphs indicate cell lines used: circles, Control-1 and *TREX1*-KO-1 pair; squares, Control-2 and *TREX1*-KO-2 pair. Colors in graphs represent the control lines (blue) or *TREX1*-KO lines (yellow). *p < 0.05.

**Table S1: Related to Fig. 2 Cholesterol synthesis is dysregulated in *TREX1*-KO microglia**

All 121 differentially expressed genes between control and *TREX1-*KO microglia by bulk RNA-seq. For each gene, table contains base-mean, log2 fold change (FC), standard error (StdErr), Wald-Stats, P-value, and P-adjusted value.

**Table S2: Related to Discussion – In search of EGFR ligands downregulated in *TREX1­-*KO microglia**

DAVID GO analysis of all differentially expressed genes which represent secreted and cell membrane markers. For each gene, table contains whether the gene codes for a secreted protein or a cell membrane protein, Log2(FC), and P-adjusted value.
